# RNA-seq for gene identification and transcript profiling in relation to root growth of bermudagrass (*Cynodon dactylon*) under salinity stress

**DOI:** 10.1186/s12864-015-1799-3

**Published:** 2015-08-04

**Authors:** Longxing Hu, Huiying Li, Liang Chen, Yanhong Lou, Erick Amombo, Jinmin Fu

**Affiliations:** Key Laboratory of Plant Germplasm Enhancement and Specialty Agriculture, Wuhan Botanical Garden, The Chinese Academy of Sciences, Wuhan, Hubei 430074 PR China

**Keywords:** *Cynodon dactylon*, Cell wall loosening, Salt stress, Transcriptome, Root growth

## Abstract

**Background:**

Soil salinity is one of the most significant abiotic stresses affecting plant shoots and roots growth. The adjustment of root architecture to spatio-temporal heterogeneity in salinity is particularly critical for plant growth and survival. Bermudagrass (*Cynodon dactylon*) is a widely used turf and forage perennial grass with a high degree of salinity tolerance. Salinity appears to stimulate the growth of roots and decrease their mortality in tolerant bermudagrass. To estimate a broad spectrum of genes related to root elongation affected by salt stress and the molecular mechanisms that control the positive response of root architecture to salinity, we analyzed the transcriptome of bermudagrass root tips in response to salinity.

**Results:**

RNA-sequencing was performed in root tips of two bermudagrass genotypes contrasting in salt tolerance. A total of 237,850,130 high quality clean reads were generated and 250,359 transcripts were assembled with an average length of 1115 bp. Totally, 103,324 unigenes obtained with 53,765 unigenes (52 %) successfully annotated in databases. Bioinformatics analysis indicated that major transcription factor (TF) families linked to stress responses and growth regulation (MYB, bHLH, WRKY) were differentially expressed in root tips of bermudagrass under salinity. In addition, genes related to cell wall loosening and stiffening (xyloglucan endotransglucosylase/hydrolases, peroxidases) were identified.

**Conclusions:**

RNA-seq analysis identified candidate genes encoding TFs involved in the regulation of lignin synthesis, reactive oxygen species (ROS) homeostasis controlled by peroxidases, and the regulation of phytohormone signaling that promote cell wall loosening and therefore root growth under salinity.

**Electronic supplementary material:**

The online version of this article (doi:10.1186/s12864-015-1799-3) contains supplementary material, which is available to authorized users.

## Background

Soil salinity is one of the most significant abiotic stresses affecting plant shoots and roots growth. Generally, a significant growth reduction in plant shoots is observed under salinity stress [1] as a result of limited photosynthesis [2]. Consequently, most morphological and transcriptional studies on the effects of salinity have majorly focused on shoots and leaves. However, the effect of salinity stress on roots should be more vivid as the root is the organ directly exposed to the salinity [3]. In addition, roots serve numerous key functions, including plant anchourage in the soil, water and nutrients absorption, hormones and metabolites synthesis [4, 5]. Plant root systems display an array of responses to saline conditions, and root architecture alteration is one of such phenotypic responses [6]. These responses are dynamically adjusted in morphology in relation to spatial and temporal salinity heterogeneity to enhance root adaptability to saline conditions [7]. Previous studies on salinity have primarily focused more on the negative effect of salinity than the positive effects on root growth. In most plants, root growth was inhibited under salinity stress [2, 8, 9]. However, for halophytes and a few salt tolerant plants species, root growth was maintained or even promoted under salinity stress. Such responses have been considered as one of the strategies for acclimation to salt stress [10, 11].

Plant roots comprise different regions involved in specific functions for the overall plant maintenance. Root tip is one of the essential regions, which encompasses the cap, the apical meristem, the cell elongation zone and the maturation zone [12]. Root tip is a region of active cell division, elongation. It is also the region of synthesizing vital phytohormones that support continuous growth and development under normal or stressed-conditions [13]. The molecular mechanisms of root growth inhibition under salinity has been extensively investigated [9, 14, 15], while little is known about the molecular mechanisms that control the physiologically well documented processes that are involved in the growth maintenance or promotion of roots in response to salinity stress.

Next generation sequencing (NGS) is a moniker used to represent multiple high throughput or massively parallel nucleic acid sequencing technologies that have emerged since the mid 2000s [16]. In contrast to traditional Sanger sequencing, where typically a few sequential or parallel sequencing reactions of relatively long read lengths (700–1000 bp) generate a modest amount of data, the shared basis of most NGS technologies is the simultaneous execution of millions of sequencing reactions of relatively short read length (30–500 bp) in parallel, and generation of gigabases (Gb) of sequence data per run [16]. The high throughput and low sequencing costs provided by NGS systems has been demonstrated that RNA sequencing is a powerful tool for comparing gene expression, discovering novel transcripts and rare transcripts in plants [17]. RNA-Seq results reveal high levels of reproducibility, for both technical and biological replicates [18]. Currently, NGS is a method of choice to unravel a diversity of stress responses on a transcriptome-wide scale in non-model plant species, where the complete genome sequence and annotation are not yet available. Using two different genotypes contrasting in their salinity stress response facilitated the coverage of a broad spectrum of genes influenced by salt stress, including those involved in a general stress response network, in susceptibility to NaCl and in salt adaptation.

The adjustment of root architecture to spatio-temporal heterogeneity in salinity is particularly crucial for plants growth and survival. Bermudagrass (*Cynodon dactylon*) is a widely used turf and forage perennial grass, and widely adapted in a number of climatic zones around the world. In addition, bermudagrass has a high degree of salinity tolerance and it is well adapted to salt spray and coastal settings. A moderate salinity appears to stimulate growth of roots and decrease their mortality in this botanical species [11]. This stimulatory effect was found to be genotype-specific. However, information about the genetic and genomic underpinnings associated with these responses is limited. Moreover, molecular mechanisms that control the positive response of root architecture to salinity are still undocumented.

In this study, we performed a transcriptome analysis using RNA-seq to analyze the transcriptome of roots focusing on the molecular basis of the physiological processes during root elongation under salinity stress in two distinct bermudagrass genotypes: C198, a salt-susceptible genotype, and C43, a salt tolerant genotype [11]. The transcriptome analysis identified a complex network of reactive oxygen species (ROS) homeostasis, cell wall loosening and transcription factors involved in the regulation of root tip elongation under salinity. In addition, the comprehensive analysis of bermudagrass root transcriptome can further be utilized as a reference to conduct future research on plant roots.

## Results and discussions

### Sequencing output and assembly

The bermudagrass salt/control RNA samples described above were used for Illumina Genome Analyzer deep sequencing. An approximate of 0.123 billion (C43) and 0.128 billion (C198) raw reads were generated, and approximately 0.116 billion and 0.121 billion clean reads were generated from C43 and C198 respectively, with a total of 0.25 billion raw reads and 0.24 billion cleans in this sequencing (Table 1). Among all the clean reads, more than 95 % had Phred-like quality scores at the Q20 level (an error probability of 1 %). The four set clean reads of the two genotypes were *de novo* assembled into one reference transcriptome with the “Trinity” program. After assembly, the four set of clean reads were mapped to the reference transcriptome. Approximately 91 million (C43) and 94 million (C198) read were mapped to the reference transcriptome, which account for 78 % and 77 % of the total clean reads for C43 and C198, respectively (Table 1).

**Table 1:** Summary of the sequencing results

Sample name	Raw reads	Clean reads	Total mapped
C43_control	57,677,570	54,390,598	42,095,182 (77.39 %)
C43_salt	65,810,400	62,197,682	48,876,470 (78.58 %)
subtotal	123,487,970	116,588,280	90,971,652 (78.0 %)
C198_control	60,527,682	57,239,830	44,344,526 (77.47 %)
C198_salt	67,794,164	64,022,020	49,449,614 (77.24 %)
Subtotal	128,321,846	121,261,850	93,794,140 (77.3 %)
total	251,809,816	237,850,130	

Root tips of two bermudagrass genotypes with differential salt tolerance, C43 (tolerant) and C198 (sensitive) were sampled for RNA sequencing after exposed to 0 (control) and 200 mM NaCl (salt) for 7 days

Using the “Trinity” assembler, totally 250,359 transcripts were generated with an average length of 1115 bp and an N50 of 1742 bp, and totally 103,324 unigenes with an average length of 764 bp and an N50 of 1311 bp were obtained by combining C43 and C198 clean reads (Table 2). The length distribution of the transcripts and unigenes from combined C43 and C198 were in the 200–500 bp range making up 35.6 % and 57.5 % of the total, respectively (Table 2).

**Table 2:** Summary of the assembly results

	Transcript length interval					Assembly					
	200-500 bp	500-1kbp	1 k-2kbp	>2kbp	total	Min Length	Mean Length	Median Length	Max Length	N50	N90
transcripts	89,217 (35.6 %)	56,313 (22.5 %)	65,276 (26.1 %)	39,553 (15.8 %)	250,359 (100 %)	201	1115	787	16458	1742	499
unigenes	59,368 (57.5 %)	20,295 (19.6 %)	15,256 (14.8 %)	8,405 (8.1 %)	103,324 (100 %)	201	764	414	16458	1311	294

The N50 value is defined as the contig length where half the assembly is represented by contigs of this size or longer; the N90 value is defined as the contig length where ninety percent of the assembly is represented by contigs of this size or longer

### Functional annotation of assembled unigenes

**Fig. 1:**
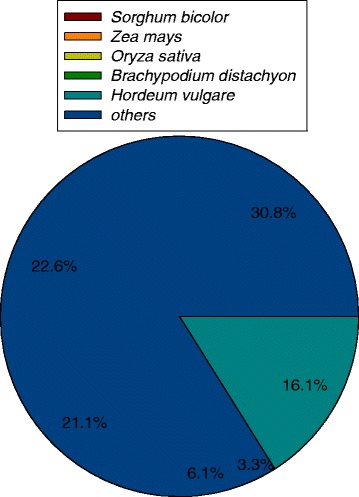
Species distribution of the top BLAST hits for the bermudagrass sequences

All the assembled high-quality unigenes were first blasted against the National Centre for Biotechnology Information (NCBI) non-redundant (NR) database using BLASTX with a cut-off E-value of 10-5 [19]. Of the 103,324 all unigenes, 45,957 (44.47 %) returned at least one match at the E-value < 10–5. 55.5 % of the unigenes did not match to known genes in the database due to the absence of genome and EST information for C. *dactylon* or closely related taxa (Table 3). In addition, all the assembled unigenes were annotated by aligning with the other six public databases, including NCBI nucleotide sequences (Nt), Protein family (Pfam), euKaryotic Ortholog Groups (KOG), a manually annotated and reviewed protein sequence database (Swiss-Prot) and Gene Ontology (GO) database with a cut-off E-value of 10^−5^. Analyses showed that 26,243 unigenes (25.4 % of all unigenes) were annotated with a significant BLAST result in the Nr database; 30,741 unigenes (29.8 % of all unigenes) were annotated in Swiss-Prot database; and 35,960 (34.8 % of all unigenes) unigenes were annotated in the Pfam, and 40,483 (39.2 %) were annotated in GO databases (Table 3). In total, there were 53,765 unigenes (52 %) successfully annotated in at least one of the NR, Nt, Swiss-Prot, Kyoto Encyclopedia of Genes and Genomes (KEGG), GO, KOG and Pfam databases, with 2936 unigenes (2.8 %) in all seven databases (Table 3). The majority of transcripts had a significant level of sequence identity to S*orghum bicolor*, *Zea mays* and *Oryza sativa* proteins, which account for 30.8 %, 22.6 % and 21.1 % of the total transcripts, respectively (Fig. 1).

**Table 3:** Summary of the annotation results.

Data base	Number of Unigenes	Percentage (%)
Annotated in NR	45957	44.5
Annotated in NT	26243	25.4
Annotated in KO	7336	7.1
Annotated in SwissProt	30741	29.8
Annotated in PFAM	35960	34.8
Annotated in GO	40483	39.2
Annotated in KOG	18003	17.4
Annotated in all Databases	2936	2.8
Annotated in at least one Database	53765	52.0
Total Unigenes	103324	100

All the assembled high-quality unigenes were annotated by aligning with the public databases, including NCBI non-redundant protein database (NR), NCBI-nucleotide sequences (Nt), Protein family (Pfam), euKaryotic Ortholog Groups (KOG), a manually annotated and reviewed protein sequence database (Swiss-Prot) and Gene Ontology (GO) database

### Gene ontology (GO) classification

**Fig. 2:**
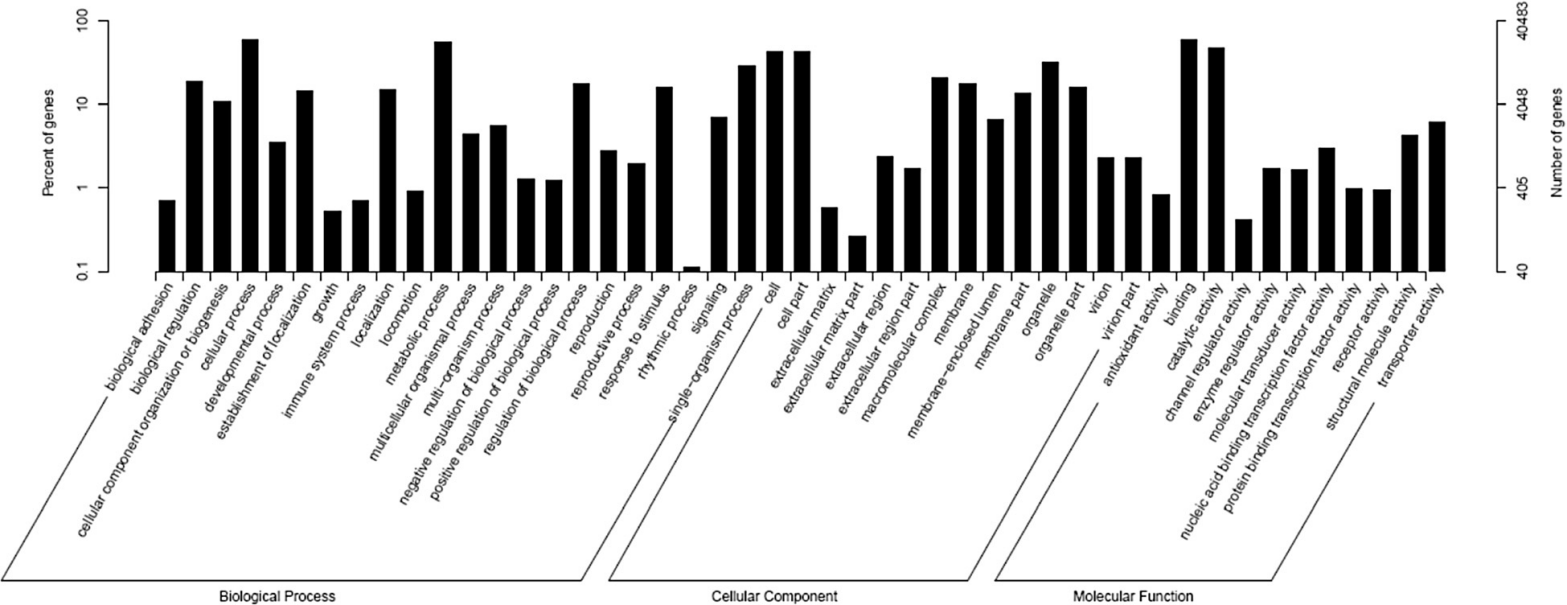
Histogram of gene ontology (GO) classification. The results are summarized in three main categories: biological process, cellular component and molecular function

Based on the sequence homology, there were 40,483 unigenes annotated into three ontologies with 47 functional groups (Fig. 2, Additional file 1). Among these groups, genes involved in ‘cellular process’ (23,647), ‘metabolic process’ (22,453) and ‘single-organism process’ (11,836) were highly represented in the biological process (BP) category. The cellular component (CC) category mainly comprised proteins involved in ‘cell’ (17,029), ‘cell part’ (17,002) and ‘organelle’ (13,006). Within the molecular function (MF) category, ‘binding’ (23,866), ‘catalytic activity’ (19,006) and ‘transporter activity’ (2521) were highly represented (Fig. 2).

**Fig. 3:**
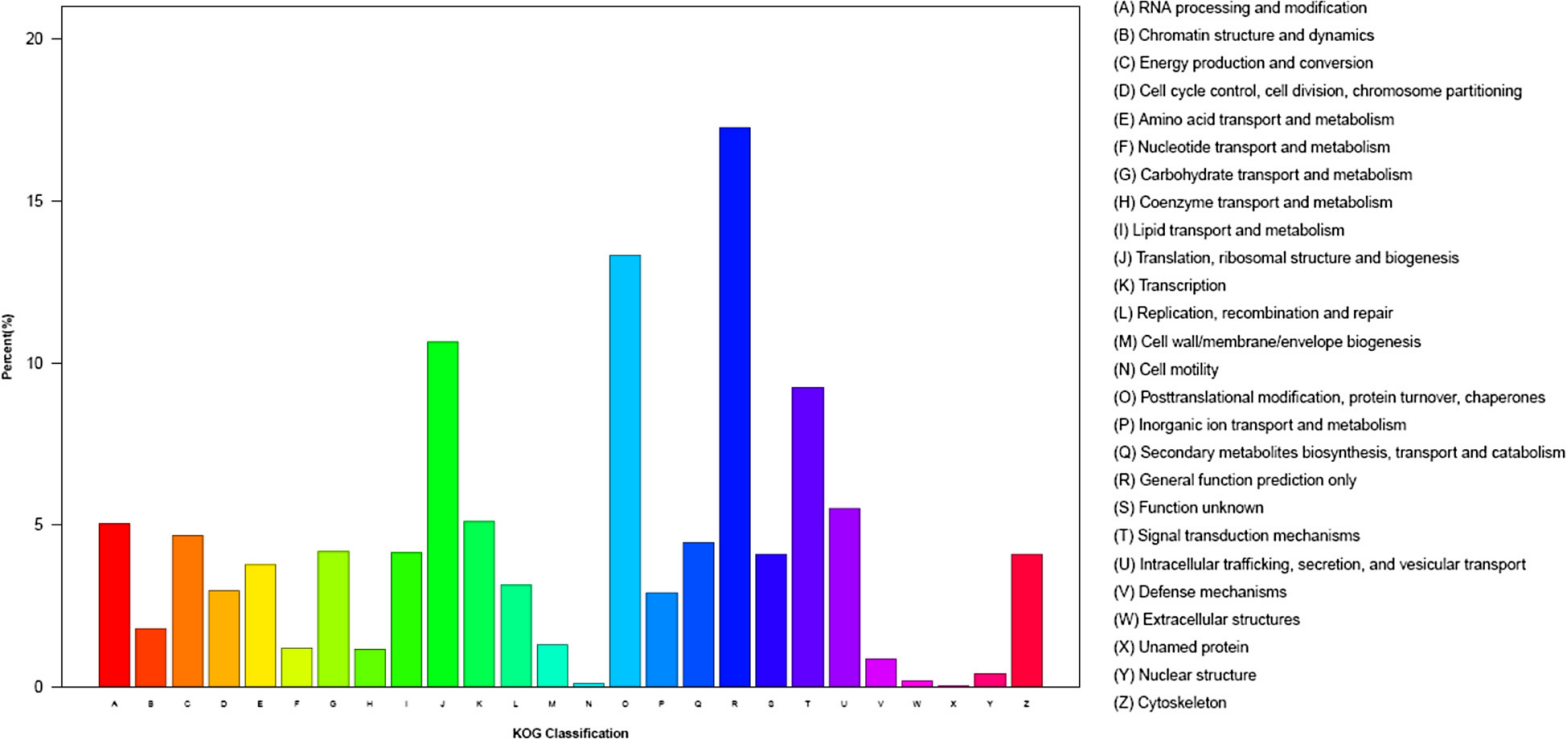
The euKaryotic Ortholog Groups (KOG) annotation of putative proteins. All 18,003 putative proteins showing significant homology to those in KOG database were functionally classified into 26 molecular families

In addition, all unigenes were subjected to KOG classification for functional prediction. Out of 45,957 nr hits, there were 18,003 unigenes assigned to KOG classification and divided into 26 specific categories (Fig. 3, Additional file 2). Among the 26 KOG categories, the ‘general functional prediction only’ (3110, 17.3 %) was the largest group, followed by post-translational modification, protein turnover, chaperon’ (2400, 13.3 %), ‘translation’ (1915, 10.6 %), ‘signal transduction’ (1664, 9.2 %). The categories ‘extracellular structures (30, 0.17 %) and ‘cell motility’ (18, 0.1 %) had the fewest corresponding genes.

**Fig. 4:**
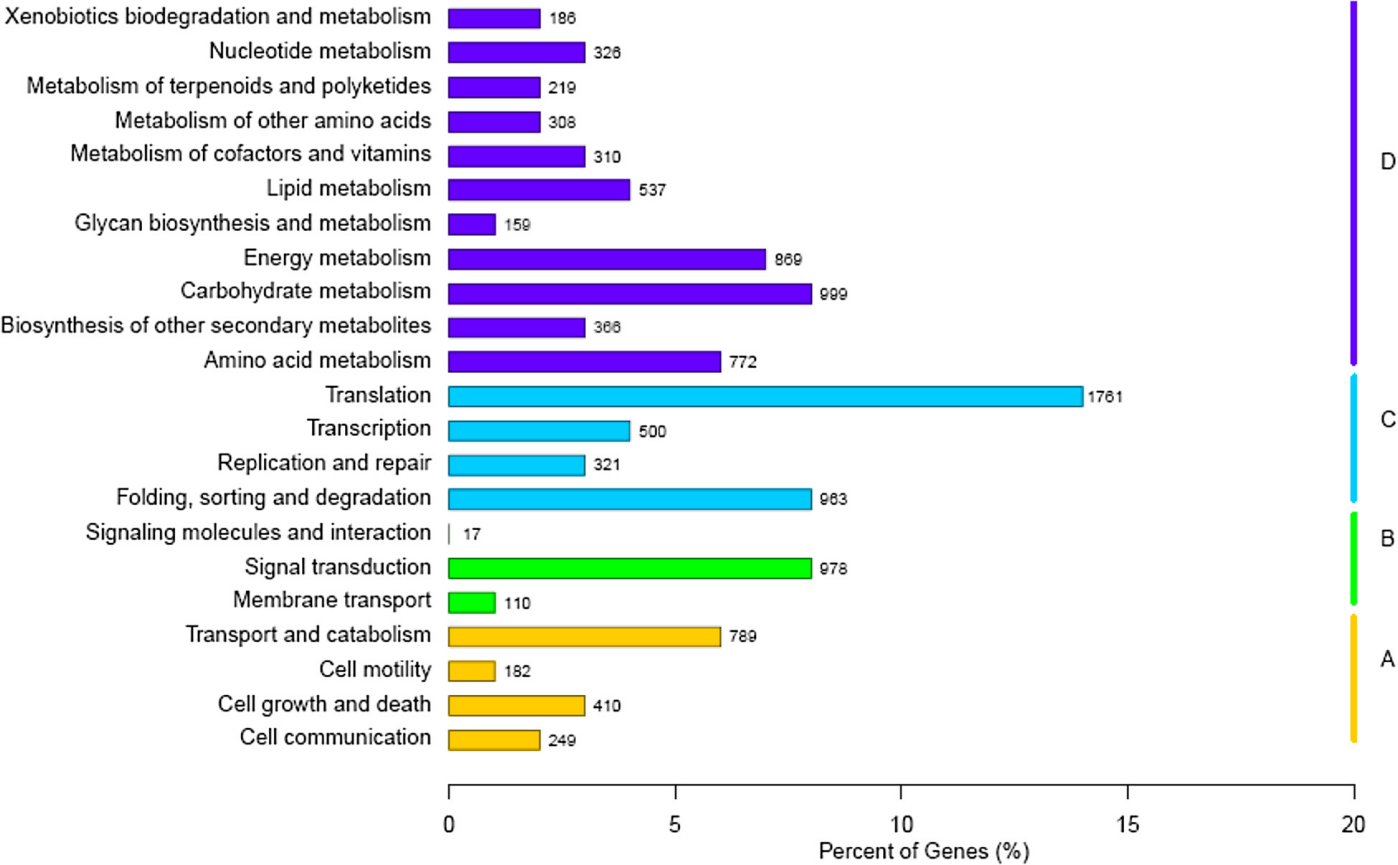
Pathway assignment based on Kyoto Encyclopedia of Genes and Genomes (KEGG). **a** Cellular Processes; **b** Environmental Information Processing; **c** Genetic Information Processing; **d** Metabolism

To identify further the active biological pathways in *C. dactylon*, the 45,957 annotated unigenes were mapped to the reference canonical pathways in the KEGG (Fig. 4, Additional file 3). Among those, 12,343 unigenes were assigned to 248 KEGG pathways (Additional file 3). The pathways involving the highest number of unique transcripts were ‘translation’ (1761 unigenes), followed by ‘carbohydrate metabolism’ (999 unigenes), ‘signal transduction’ (978 unigenes), ‘folding, sorting and degradation’ (963 unigenes) and ‘energy metabolism’ (869 unigenes), indicating that these were the active pathways in *C. dactylon*.

### Differential expression analysis of assembled transcripts

**Fig. 5:**
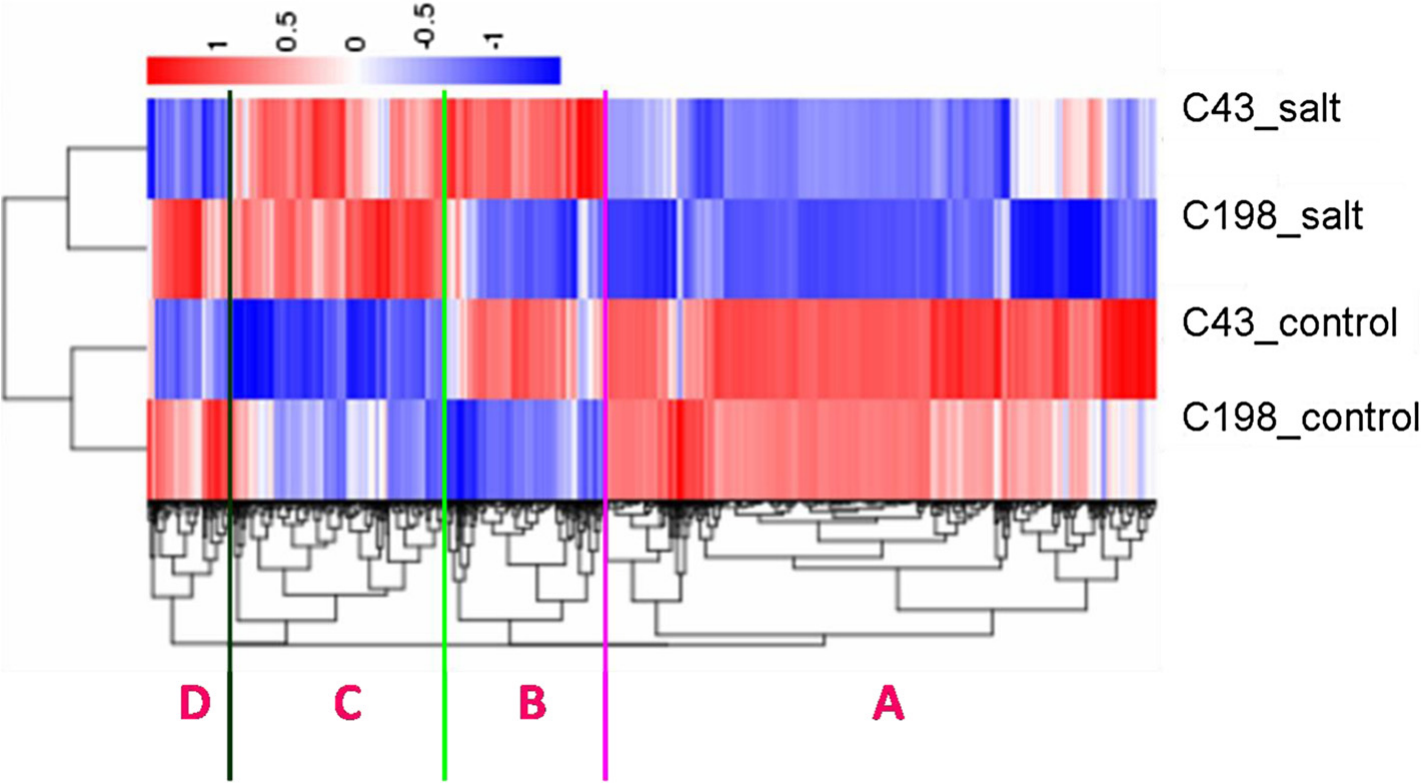
Hierarchical clustering analysis of salinity-induced changes in gene expression in root tips of bermudagrass (C43_salt, 200 mM NaCl treated C43; C198_salt, 200 mM NaCl treated C198; C43_control, 0 mM NaCl treated C43; C198_control, 0 mM NaCl treated C198)

We used the normalized RPKM (reads per kilobase per million) to quantify the transcript level in reads, which facilitated the comparison of mRNA levels both within and between genotypes. Differential expressed genes (DEGs) (q-value < 0.005 and |log_2_ (fold change)| >1) were defined as genes that were significantly enriched or depleted in one genotype and/or treatment relative to the other genotype and/or treatment (Additional file 4). A hierarchical clustering of the differentially expressed genes based on the four sample’s log10RPKM was made, so we could observe the overall gene expression pattern. The blue bands identify low quantity gene expression, while the red represent the high quantity gene expression (Fig. 5). Four groups of DEGs with specific expression patterns were delineated from the clustering. DEGs showed down-regulation in Group A and up-regulation in group C under salinity for both genotypes when compared to the control level. DEGs in Group B and group D showed no changes in expression levels between control and salinity in both genotypes with contrary expression pattern between the two genotypes (Fig. 5).

**Fig. 6:**
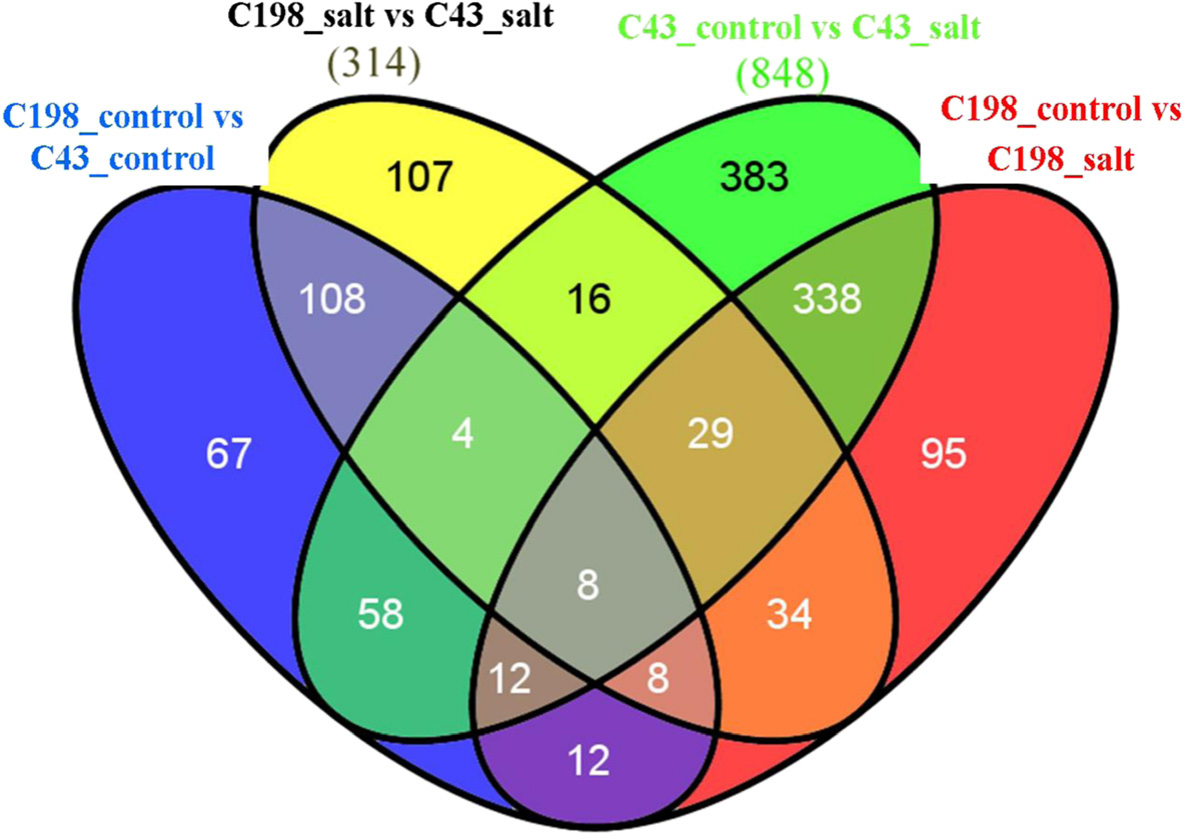
Comparison between the amounts of differential expressed genes (DEGs) found in the two genotypes by mapping reads to the de novo assembled TCs. A Venn diagram depicting the number of statistically significant (>2-fold) DEGs when the de novo transcriptome (green and red ovals, FDR <0.025). The numbers of DEGs exclusively expressed in one sample are shown in each circle. The numbers of DEGs with a common or opposite tendency of expression changes between the two treatments are shown in the overlapping regions. The total numbers of DEGs in each treatment are shown outside the circles (C43_salt, 200 mM NaCl treated C43; C198_salt, 200 mM NaCl treated C198; C43_control, 0 mM NaCl treated C43; C198_control, 0 mM NaCl treated C198)

Comparison of changes in gene expression between control and salt-stressed plants in two genotypes had shown similarities and considerable differences. The Venn diagram indicates the distribution of expressed genes among the four samples. Under control (0 mM NaCl) and salt stress (200 mM NaCl), there were 277 and 314 DEGs between C43 and C198, respectively. Under salt-stressed conditions, there were 848 and 536 DEGs between the control and the salt-stressed roots for C43 and C198, respectively (Fig. 6). There were more genes exclusively differentially expressed in C43 (383) than that of C198 (95) under salinity.

### Validate the DEGs by real-time RT-PCR analysis

**Fig. 7:**
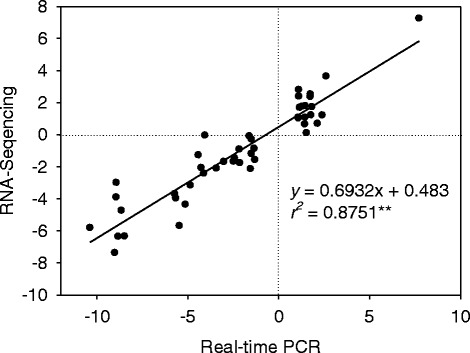
qRT-PCR validations of differentially expressed genes in root of bermudagrass under salinity. Correlation of fold change analyzed by RNA-Seq platform (x axis) with data obtained using real-time PCR (y axis)

To validate the data from RNA-sequencing, 46 DEGs were randomly selected for real-time RT-PCR analysis in both genotypes in response to salt stress. The primers of selected genes are listed in Additional file 5. Actin was used as reference gene for data normalization according to Hu et al. [11]. The qRT-PCR results showed a strong correlation with the RNA-seq-generated data (Pearson correlation coefficients r = 0.87; Fig. 7, Additional file 5).

### Transcription factors in relation to salinity stress and root growth regulation

The assembled transcriptome of salt-stressed root tips demonstrated that totally 24 unique TFs were differentially expressed under salinity stress in both genotypes, with 17 exclusively in C43 (Table 4, Additional file 6). They included major TF families linked to stress responses and growth regulation, such as AP2/ERF, bZIP, MYB, MYC, NAC, MADS-box, WRKY, bHLH, zinc finger family (Table 4). Several salt-induced TF genes also respond to other abiotic stresses such as osmotic stress, cold and heat [15], suggesting that they generally participate in stress response, and the spatial differences of TF gene regulation by environmental stresses in root tips may be crucial for the adaptation of their growth to specific soil environments. TF genes encoding AP2/ERF, MYB, MYB, NAC, WRKY showed a considerable enhancement of expression in root apexes in response to salt stress when compared to whole roots in *M. truncatula* [15]*.* The regulation of specific members of TF families in Medicago root tips supports the hypothesis that these genes may intersect root developmental pathways and salt-related transcriptional networks [15].

**Table 4:** Transcription factors differentially expressed in the two genotypes under salinity stress

Transcription factors family	C43	C198
AP2/ERF	1	0
bZIP	1	1
GATA5	1	0
MADS-box	3	2
MYB	3	1
MYC	1	0
NAC	1	0
bHLH92	1	0
WRKY	6	1
Zinc finger	4	1
RING-H2 finger protein ATL65	0	1
RING finger and CHY zinc finger (RCHY1)	1	0
total	23	7

Among the differentially expressed TF genes in root tip, there were several classes (MYB, NAC and bHLH) involved in growth regulation, and other metabolic processes. The differential expression of many of these genes in the root tip is not striking as they participate in active processes in the root tip such as expansion and cell division, as well as the encountering a variety of biotic/abiotic stresses [19]. It has been previously demonstrated that bHLH-type TFs are linked to the adaptation of *Medicago truncatula* to saline soil environments [20], and a group of bHLH-type TFs were found to be involved in the root growth and development of *Medicago sativa* under salinity [15]. A bHLH-type TF (comp117508_c0) was up-regulated, while a number of peroxidases were down-regulated in C43 genotype, this observation indicated that the bHLH TF might modulate the ROS balance by directly regulating the expression of a set of peroxidases, consequently, regulating the root cell proliferation and differentiation [21].

The MYB TFs is one of the largest superfamily of plant TFs. Apart from metabolic, signal transduction and defense-related pathways, the induced or repressed expressions of TFs affected plant growth and development under salinity [22, 23]. The over expression of *MYB31* and *MYB42* repressed the expression of genes related to lignin synthesis in maize [24], which exhibited up to 45 % reduction in lignin content and substantially increased leaf, root, and stem growth [25]. A MYB-like TF (KUA1) modulates leaf cell expansion and final organ size by controlling the expression of peroxidases and ROS homeostasis [26]. Three MYB TFs were differentially expressed in C43 with two down-regulated (comp134391_c0, comp112426_c0) and one MYB TF (comp120905_c3) up-regulated under salinity. This observation suggests that the induction or repression of MYB TFs may be participating in the lignin synthesis and/or ROS homeostasis controlled by peroxidases.

The WRKY transcription factors are considered to be repressors of the GA signaling pathway [27], activators of the ABA signaling pathway [28] and regulators of many other signaling pathways in plants [29]. *WRKY31* gene was found to enhance disease resistance and reduced lateral root formation and elongation with induced constitutive expression of auxin-response genes, such as *OsIAA4* and *OsCrl1* genes [30]. Two WRKY TFs (comp115018_c1 and comp120384_c0) were down-regulated and three WRKY TFs (comp112466_c0, comp122277_c0 and comp117715_c0) up-regulated in C43. One WRKY TFs (comp123500_c0) were up-regulated in C198 (Additional file 6). These differentially expressed WRKY TFs may be involved in the regulation of root growth under salinity through the regulation of phytohormone signaling.

#### The expression pattern of genes involved in cell wall loosening

In contrast to mammalian cells, plant cells are encased by a cell wall that gives structural support, and cell expansion is affected by alterations in cell wall and architecture [31]. Several proteins have been directly implicated in cell wall loosening, including xyloglucan endotransglucosylase/hydrolases (XETs) [32]. In addition, non-enzymatic processes involving ROS that produce wall polysaccharide scission also participate in cell wall loosening [33]. Cell walls as conditioners of cell growth under salt stress have been investigated in detail [34]. The balance between cell wall loosening and stiffening activities defines the regions of accelerated and decelerated root growth in the elongation zone [35]. The root system comprises different regions that are involved in specific functions for the overall plant sustenance. Root tips are essential regions that encompass the root apical meristem and elongation zone. They produce pall types of cells in a highly defined pattern of cell division to assist growth during saline conditions [36].

Root tip transcriptome analyses indicate that the expression of genes related to cell wall loosening and stiffening is modified by salinity in bermudagrass. One XET transcript (comp116615_c0) was found to be up-regulated in C43 (Additional file 7). The XET transcript expression pattern following the distribution of growth rates in the growing zone of *Festuca pratensis*, and the resulting XET activity was proposed to be involved in cell wall modification processes during cell elongation [37]. XET activity was enhanced in the apical region of maize roots from plants grown under low water potential [38]. These results indicated that the increased expression level of XET transcript in the root tips of bermudagrass under salinity might be necessary for maintaining root elongation under these conditions.

Salinity may promote cell wall stiffening possibly mediated by peroxidase [39]. Peroxidases are considered bifunctional enzymes that not only oxidize various substrates in the presence of H_2_O_2_, but also generate H_2_O_2_ [40]. The relationship between plant peroxidase and cell expansion restriction has been shown in several studies [41]. Apoplastic peroxidases are known to either restrict or promote cell expansion [21]. In *C. gayana* leaves, the peroxidase activity increase, and the phenolic compounds of the cell walls caused a reduction in the length of the elongation zone grown under saline conditions [42]. However, Cramer et al. [43] did not observe increased wall stiffening or stimulated peroxidase activity under salinity. This result suggests that the apparent contradictory effects are linked to the regulatory modes under which the peroxidases are working. The repressed expression of peroxidases by KUA1 (a MYB-like TFs) in leaves promoted cell expansion, which is clearly linked to changed levels of apoplastic H_2_O_2_ [26]. In our study, 21 peroxidases transcript were found to be differentially expressed with 19 down-regulated in C43 and 17 down-regulated in C198. The down-regulation of those peroxidases transcript may favor root elongation by the reduction of apoplastic H_2_O_2_, and the generation of oxygen radicals to cleave the cell wall polymers thus promote cell wall loosening and therefore root growth [26].

## Conclusions

Root growth maintenance or promotion under salinity is a complex process that integrates spatio-temporal developmental events from the sensing of osmotic and ionic stress. We performed RNA-seq in the root tips of two genotypes of bermudagrass, which had different root growth characters and salt tolerance. The aim of the sequencing was to identify the causes for the observed changes in the spatial pattern of root elongation under salinity. In total, 250,359 transcripts with an average length of 1115 bp and totally 103,324 unigenes obtained with 53,765 unigenes (52 %) were successfully annotated in databases. Moreover, the 848 and 536 differentially expressed unigenes in C43 and C198 were useful to identify the genes related to the root growth regulation under salinity. The RNA-seq identified candidate genes encoding TFs involved in the regulation of lignin synthesis, ROS homeostasis controlled by peroxidases, and the regulation of phytohormone signaling that promote cell wall loosening and hence root growth under salinity. Our results support and add detailed molecular information to the root growth maintenance under stress via increased wall loosening. The data suggest need for control of intracellular ROS content by peroxidase under the regulation of transcription factors for apoplastic hydrogen peroxide production and other cell wall proteins for wall loosening induction. Collectively, the data indicate that the regulation of root growth under salinity involves changes in many different aspects of cell metabolism, signaling, and transport.

## Methods

### Plant growth, salt treatment and sampling

Uniform stolons (5 cm long) of bermudagrass, ‘C43’ (salt tolerant) and ‘C198’ (salt sensitive) were planted in solid growth substances (peat soil: sand = 2:1, v/v) [11]. After 14 d of establishment, equal amount of plantlets were transfer to plastic pots (7 cm diameter and 9 cm height) filled with coarse silica sand as the plant anchor medium. Pots were suspended over tubs containing 46 L of constantly aerated half-strength Hoagland’s solution [44]. The tubs were refilled every other day and renewed weekly. Pot bottoms consisted of a coarse nylon screen allowing roots to freely grow into the solutions. Plants were grown in an environmentally controlled walk-in growth room with the temperature regime of 30 /25 °C (day/night), photosynthetically active radiation (*PAR*) levels of 800 μmol · m^−2^ · s^−1^ at canopy height for 14 h. Plants were allowed to adapt to this nutrient solution for 2 weeks. During this period, the plant shoots were hand-clipped weekly at 6 cm height, and roots were clipped back to the bottoms of the pots at the beginning of the salt treatment in order to allow the plants to reach full maturity and develop uniform and equal size roots and shoots.

After 2 weeks of hydroculture, the half-strength Hoagland’s solution was supplemented with 200 mM NaCl. After 7 d of treatment, plants were removed from the nutrient solution and gently washed for 20 s with distilled water for the roots. Roots elongated from nylon screen into nutrient solution were sampled after 4 h light. The root tips, encompassing the meristem and the elongation zone, were excised with a scalpel from the remaining root system, and immediately frozen in liquid nitrogen. Multiple independent biological replicates, each containing a pool of twenty different plants, were sampled for mRNA-Seq (two biological replicates) and reverse transcription-PCR (RT-PCR) or quantitative RT-PCR (qRT-PCR) validation (three biological replicates).

The salt treatments and grass genotypes were arranged in a randomized complete block design with six replicates. Physiological measuring to one set of plants and taking tissue samples for RNA isolation and metabolite analysis to another set of plants were performed simultaneously at the time period of 1000–1500 h.

### Total RNA, mRNA isolation and library preparation for transcriptome sequencing

Total RNA was extracted using Trizol reagent (Invitrogen) and purified using the RNeasy Plant Mini kit (Qiagen) according to the Handbook, with an additional sonication step after addition of RLT buffer (Qiagen). The quality and integrity of RNA was checked by Agilent Bioanalyzer 2100 system (Agilent Technologies, CA, USA) and agarose gel electrophoresis. A total amount of 3 μg RNA per sample from root tips was used for mRNA-Seq library construction using NEBNext® Ultra™ RNA Library Prep Kit for Illumina® (NEB, USA) following manufacturer’s recommendations. The mRNA was isolated using oligo(dT)-attached magnetic beads and subsequently fragmented using divalent cations under elevated temperature, and the cleaved RNA fragments were copied into first-strand cDNA using random hexamer primer and M-MuLV Reverse Transcriptase. Second strand cDNA synthesis was subsequently performed using DNA Polymerase I and RNase H, and the cDNA fragment were processed for end repair, an addition of a single “A” base, and ligation of the adapters. After second-strand cDNA synthesis and adaptor ligation, cDNA fragments of 150 ~ 200 bp in length were isolated with AMPure XP system (Beckman Coulter, Beverly, USA). These products were then purified and enriched by PCR to create the final cDNA library. After cluster generation on a cBot Cluster Generation System using TruSeq PE Cluster Kit v3-cBot-HS (Illumia), the multiplexed library was sequenced on an Illumina Hiseq 2000 platform according to the manufacturer’s recommendations (Illumina) at Novogene Bioinformatics Institute, Beijing, China. RNA-seq read data were deposited to the NCBI Sequence Read Archive (NCBI SRA) under accession number SRR2075766.

### Preprocessing and *de novo* assembly

In total, 25.18 G bases (Gb) raw reads were generated by Illumina Hi-seq platform, with a total of ~ 5.77 Gb, ~6.05, ~ 6.58 Gb and ~ 6.78 Gb in C43-control, C198-control, C43-salt and C198-salt, respectively. The raw reads were initially processed through in-house perl scripts to remove the adapter sequences, reads containing ploy-N, and low-quality bases, and finally get the clean data (clean reads). All the downstream analyses were based on clean data with high quality. After preprocessing, we obtained four set clean reads, with a total of ~ 5.44 Gb, ~ 5.72 Gb, ~6.22 Gb and ~6.4 Gb quality filtered short reads for C43-control, C198-control, C43-salt and C198-salt, respectively. The four set clean reads of the two species were de novo assembled with the “Trinity” program (v2012-10-05) after merged following the protocol documented in [45] with the min_kmer_cov set to 2 and all other parameters set default. The base calling and base quality assignment were evaluated by using PHRED [46].

### Unigene annotation and classification

Unigenes (≥100 bp) were used to search against the NR [19], SwissProt [47], KEGG (version 58) [48] and KOG [49] databases by BLASTALL package (release 2.2.22) with the significant threshold of E-value ≤ 10^−5^. Each known gene from the best BLASTx hit was parsed and assigned. The ORF of assembled transcripts was determined based on the results of BLASTx search in the following order: NR, KEGG and KOG. Extending from both sides of the aligned region, the coding region sequences were translated into amino acid sequences with the standard codon table using custom PERL scripts. For unigenes that did not align to any of the above databases were scanned by ESTScan to determine the nucleotide and amino acid sequences of the coding regions. The predicted amino sequences were submitted to search against the Pfam database (version 26.0) [50] for domain/family annotation using HMMER 3.0, with the ‘Best Match Cascade’ protocol. Gene ontology (GO) [51] terms for each unigenes were assigned based on the best BLASTx hit from the NR and Pfam database using Blast2GO software (version 2.5) [52] with an E-value threshold of 10^−5^.

### Quantification of gene expression levels and differential expression analysis

Gene expression levels were estimated by RSEM [53] for each sample. Clean data were mapped back onto the assembled transcriptome. Readcount for each gene was obtained from the mapping results and normalized to reads per kb of exon model per million mapped reads (RPKM) [53]. Prior to differential gene expression analysis, for each sequenced library, the read counts were adjusted by edgeR program package through one scaling normalized factor [54]. Differential expression analysis of two samples was performed using the DEGseq (2010) R package. P-value was adjusted using q value. q value < 0.005 and the absolute value of log2 > 1 was set as the threshold for significant difference in gene expression. Gene ontology (GO) analysis of the differentially expressed genes (DEGs) was done for biological process, cellular components and molecular function by BGI WEGO (Web Gene Ontology Annotation Plotting, http://wego.genomics.org.cn/cgi-bin/wego/index.pl) [55] and agriGO (GO Analysis Toolkit and Database for Agricultural Community, http://bioinfo.cau.edu.cn/agriGO/index.php) [56], and pathways that were statistically significantly (Q-value < 0.05) were enriched with KEGG [57]. The differential expression profiles of the genes have been colored based on the Venn diagram [58].

### Quantitative real-time PCR analysis

The expression of selected candidate genes was validated by quantitative real time PCR (qRT-PCR) using the same RNA samples as in the RNA-seq library construction. The first strand cDNA fragments were synthesized from 2 μg of total RNA using oligo(dT)_12–18_ primer using cDNA synthesis kit (Fermentas, Burlington, Ontario, Canada) according to the user manual. Gene-specific primers (Table 1) were designed based on the target gene sequences using Primer 5 software. *UBI* gene was used as internal standard. The *q*RT-PCRs were performed with ABI7500 in a final volume of 20 μL, with each containing 2 μL of cDNA, 10 μL of 2 × SYBR Green *q*PCR Mix (Takara, Otsu, Shiga, Japan) and 2 μM of the forward and reverse primers. Three independent biological replicates of each sample and two technical replicates of each biological replicate were used for real-time PCR analysis. The thermal cycling conditions were as follows: 40 cycles of 95 °C denaturation for 5 s, and 52 ~ 55 °C annealing and extension for 20 s. After the PCR, a melting curve was generated by gradually increasing the temperature to 95 °C to test the amplicon specificity. To determine relative fold differences for each sample, the CT value for each gene was normalized to the CT value for the reference gene and was calculated relative to a calibrator using the DDCT method as described by Livak and Schmittgen [59].

## Additional files

Additional file 1: 
**GO_classification.** (XLS 10826 kb) Additional file 2: 
**KOG_classification.** (XLS 270 kb) Additional file 3: 
**KEGG_classification.** (XLS 264 kb) Additional file 4: 
**Differentially expressed genes in two genotypes of bermudagrass.** (XLS 1612 kb) Additional file 5: 
**Pimers for qRT-PCR analysis.** (XLS 31 kb) Additional file 6: 
**Selected differentially expressed transcription factors.** (XLS 20 kb) Additional file 7: 
**Selected cell wall related DEGs.** (XLS 25 kb)

## Abbreviations

BLAST: Basic local alignment search tool
bHLH: Basic Helix-Loop-Helix
bZIP: Basic leucine zipper
BP: Biological process
CC: Cellular component
DEGs: Differential expressed genes
GO: Gene ontology
KEGG: The Kyoto encyclopedia of genes and genomes database
KOG: euKaryotic Ortholog Groups
MF: Molecular function
NAC: NAM/ATAF/CUC domain protein
NCBI: National center for biotechnology information
NGS: Next-generation sequencing
NR: NCBI non-redundant protein database
Nt: Nucleotide sequences database
PAR: Photosynthetically active radiation
PERL: Practical extraction and report language
Pfam: Protein family, Swiss-Prot, a manually annotated and reviewed protein sequence database
q-PCR: Real-time quantitative PCR
RNA-Seq: RNA-sequencing
ROS: Reactive oxygen species
RPKM: Reads per kb per million reads
TF: Transcription factor
XET: Xyloglucan endotransglucosylase
